# Human maternal heritage in Andalusia (Spain): its composition reveals high internal complexity and distinctive influences of mtDNA haplogroups U6 and L in the western and eastern side of region

**DOI:** 10.1186/1471-2156-15-11

**Published:** 2014-01-24

**Authors:** Candela L Hernández, Guillermo Reales, Jean-Michel Dugoujon, Andrea Novelletto, Juan Nicolás Rodríguez, Pedro Cuesta, Rosario Calderón

**Affiliations:** 1 Departamento de Zoología y Antropología Física, Facultad de Biología, Universidad Complutense, Madrid, Spain; 2 CNRS UMR 5288 Laboratoire d’Anthropologie Moléculaire et d’Imagerie de Synthèse (AMIS), Université Paul Sabatier Toulouse III, 31073 Toulouse, France; 3 Dipartimento di Biologia, Università Tor Vergata di Rome, Rome, Italy; 4 Servicio de Hematología, Hospital Juan Ramón Jiménez, Huelva, Spain; 5 Centro de Proceso de Datos, Universidad Complutense, Madrid, Spain

**Keywords:** Iberian Peninsula, Gene flow, Mediterranean space, mtDNA haplogroups U6 and L, European profiles

## Abstract

**Background:**

The archeology and history of the ancient Mediterranean have shown that this sea has been a permeable obstacle to human migration. Multiple cultural exchanges around the Mediterranean have taken place with presumably population admixtures. A gravitational territory of those migrations has been the Iberian Peninsula. Here we present a comprehensive analysis of the maternal gene pool, by means of control region sequencing and PCR-RFLP typing, of autochthonous Andalusians originating from the coastal provinces of Huelva and Granada, located respectively in the west and the east of the region.

**Results:**

The mtDNA haplogroup composition of these two southern Spanish populations has revealed a wide spectrum of haplogroups from different geographical origins. The registered frequencies of Eurasian markers, together with the high incidence and diversification of African maternal lineages (15% of the total mitochondrial variability) among Huelva Andalusians when compared to its eastwards relatives of Granada and other Iberian populations, constitute relevant findings unknown up-to-date on the characteristics of mtDNA within Andalusia that testifies a female population substructure. Therefore, Andalusia must not be considered a single, unique population.

**Conclusions:**

The maternal legacy among Andalusians reflects distinctive local histories, pointing out the role of the westernmost territory of Peninsular Spain as a noticeable recipient of multiple and diverse human migrations. The obtained results underline the necessity of further research on genetic relationships in both sides of the western Mediterranean, using carefully collected samples from autochthonous individuals. Many studies have focused on recent North African gene flow towards Iberia, yet scientific attention should be now directed to thoroughly study the introduction of European genes in northwest Africa across the sea, in order to determine its magnitude, timescale and methods, and to compare them to those terrestrial movements from eastern Africa and southwestern Asia.

## Background

The archeology and history of the ancient Mediterranean have shown that this sea has always been a permeable obstacle to human migration. Throughout prehistory onwards, people have traveled and moved across the Mediterranean and migrated in many directions. Scenarios of early human movements in the Mediterranean both over land and by sea are found in the obsidian trade of Melos island with Greece mainland (~8,000 BC), and evidences of seafaring and island occupations as Cyprus, Corsica, Sardinia and Majorca are dated ~8,500 BC [1,2]. Consequently, multiple cultural exchanges around the Mediterranean have taken place with presumably population admixtures. A critical region of those migrations has been the Iberian Peninsula. Iberia has a strategic geographical position, in the southwestern extreme of Europe, being in maritime contact with the Atlantic Ocean and Mediterranean Sea and further acting as the closest link between Africa and Europe across the narrow Strait of Gibraltar (14 km). Moreover, its extensive territory of complex relief with a markedly variation among their different regions [3], its intricate history, archeological richness and diverse and persistent socio-cultural patterns, justifies the growing scientific interest in knowing the extent of the genetic diversity of contemporary Iberian populations and the impact of migrations on their gene pool ([4-10], among others). The Cantabrian fringe of northern Iberia along with the east of Andalusia region and the Spanish Levant would have had an important role as refugee areas during the Last Glacial Maximum (LGM, ~20 thousand years ago, kya) as well as a source of a posterior European resettlement with the improvement of climatic conditions [11,12].

The mitochondrial (mtDNA) genome is being one of the most widely used molecular tools in the knowledge of Iberian genetic diversity, commonly by the joint analysis of control region and some coding region variants. Even so, in the last years, the level of phylogenetic resolution in the study of mtDNA in Iberian populations is reaching the maximum by means of complete genome sequencing, paying special attention to particular maternal lineages with different ancestral roots in diverse continental areas [7,10,13-17].

Within Peninsular Spain, the most thoroughly examined region in terms of human mtDNA genetic variation has been the Cantabrian cornice –in particular Galicia and the Basque area [18-21]. Galicians and Basques both share high values of mtDNA lineage H, and specifically of sub-clades H1 and H3, which reach frequency peaks in those territories.

Given the abundance of surveys dealing with the extent of genetic diversity both in Iberia and its islands (e.g. the Canary Islands), the absence of an exhaustive and monographic analysis addressed to know mtDNA profiles in locally defined Andalusian coastal populations is striking. The attractive prehistory and history of Andalusia, the widest and most populated region of Spain, makes its present-day human population a prominent objective to detect scenarios of population substructure and to examine the expected impact of African and other Mediterranean populations on the Iberian gene pool. Consistently with the geographical proximity between southern Spain and Africa, previous published studies have obtained results that show evidences of African-linked mtDNA lineages among Andalusians as well as high levels of diversity, either analyzing regional general samples [22-24] or focusing on other inland territories within the region [16].

**Figure 1:**
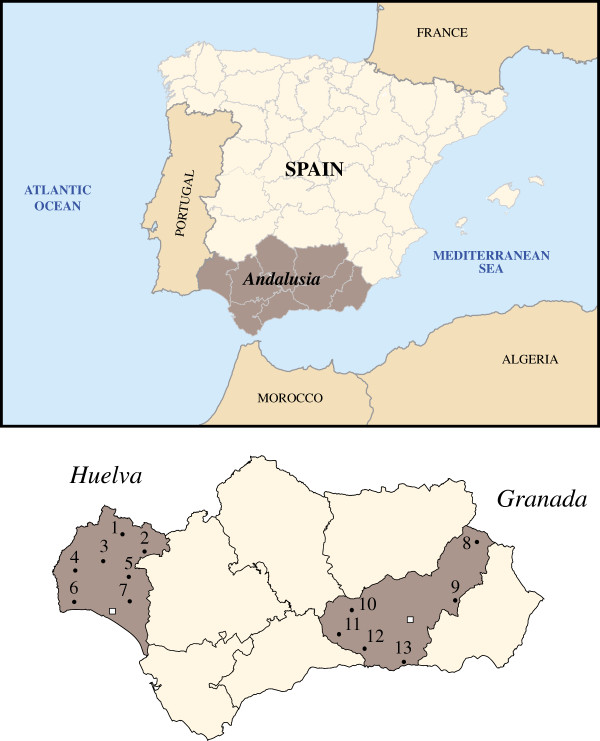
**Geographic location of the Andalusia region in the Iberian Peninsula.** The two studied Andalusian subpopulations are highlighted in dark brown. Municipalities, marked with numbers correspond to sample locations in Huelva and Granada provinces, and they were: 1: El Repilado, 2: Aracena, 3: El Cerro del Andévalo, 4: La Puebla de Guzmán, 5: Valverde del Camino, 6: Villablanca, 7: Niebla, 8: Huéscar, 9: Baza, 10: Montefrío, 11: Loja, 12: Alhama de Granada, 13: Órgiva. The capital cities of the two provinces are denoted with a white square.

In this paper, we provide novel data on mtDNA haplogroup (Hg) composition in autochthonous individuals from Huelva and Granada provinces, located respectively in the west and in the east of Andalusia region (southern Spain, see Figure 1). Both provinces are open to the sea and therefore to maritime contacts. Huelva, with a strategic Atlantic location near the Strait of Gibraltar and the Guadalquivir estuary –rich in mineral resources– would have experienced an intense commercial activity since ancient times, involving long-distance trade in metals –copper, tin and silver– as main deposits of the Tinto river area.

Huelva and Granada have had different and well-defined histories. The former is mainly characterized by the *Tartessian* culture (ca. 800–540 BC) whereas the latter is strongly linked to the *Nazari* kingdom, the Islam’s last possession in Iberia. *Tartessos* was an essential axis in Iberian protohistory, the foyer through which the Iberian Peninsula entered fully into Mediterranean history [25].

In terms of historical population dynamics, the province of Huelva has secularly registered lower demographic size than its counterpart of Granada. In the *census of Floridablanca* (1787), the first modern Spanish population record, the population of Huelva represented the 6.4% and Granada the 14.6% of Andalusia, and that ratio has been maintained in subsequent historical series. Nowadays, their population densities are 51.6 and 73.0 inhabitants per km^2^, respectively (*Instituto Nacional de Estadística*, INE 2012).

Some recent studies focused on high-resolution phylogenetic analyses of Y-chromosome haplogroups E and J, performed by this team on the same stock of Andalusian samples, have revealed that both Huelva and Granada have integrated multiple migrations and that the sources of gene flow appear to be more intense and diverse in the former than in the latter. The presence and frequency in the region of E-M81, commonly referred to as the “Berber marker”, the occurrence of E-M34, prevalent among Jews, and the appreciable representation of other Mediterranean paternal lineages (E-V13, J1-M267, and J2b-M12) would be adding further support to the role of Andalusia as an open door to human population movements, mainly across the Mediterranean, and with special intensity from Neolithic until historic times [8,9].

Guided by these and other previously published data on haploid DNA polymorphisms in current Iberian populations, the main goal addressing the present work is to evaluate the mtDNA haplogroup composition in two samples of autochthonous Andalusians. As far as we know, no investigations on this topic have provided results involving both territories and populations. Our mitochondrial data presented here will give stronger evidences for a more visible African influence in the west than in the east of Andalusia. In addition, we report phylogenetic networks based on mtDNA haplogroups U6 and L of Andalusians, other Iberian and northwest African population samples to detect gene flow on both sides of the Gibraltar Strait, and furthermore we interpret other mitochondrial outstanding features present in the current maternal composition of southern Iberians. With all these new genetic data we attempt to shed new clues about migration and peopling processes occurred in the Iberian Peninsula since ancient times onwards.

## Results

### MtDNA sequence diversity in Andalusians

**Figure 2:**
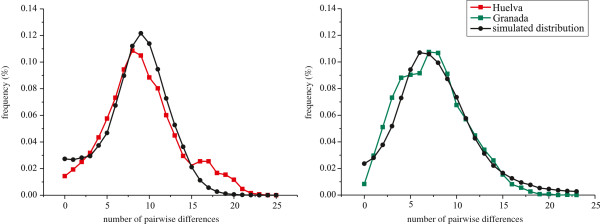
Observed and expected mismatch distributions for Huelva and Granada mtDNA control region sequences.

We have found a total of 197 different haplotypes (based on control region sequences and coding region SNPs) among 279 mtDNA sequences of autochthonous Andalusians. Few haplotypes (11/279) were shared between our two analyzed samples (further details in Additional file 1). Taking into account only control region information, we detected 104 different sequences out of 158 in Huelva (65.8%) and 95 out of 121 in Granada (78.5%). The number of singleton haplotypes has been 77, 80 and 147 in Huelva, Granada and both provinces, respectively. In all three cases, the distributions of repeated haplotypes (abundance-occurrence) fit well a power law with exponents varying between -2.12 and -3.03 and coefficients of determination (R^2^) of 0.86 and 0.91. Parameters corresponding to mtDNA sequence analysis are presented in Table 1. Estimated gene diversity values (*H*) were similar in western and eastern Andalusians, but a little higher to that found in other mainland Iberian populations (0.95-0.96) [7,16]. Tajima’s (*D*) and Fu’s *(F*_*s*_) neutrality tests showed significantly negative values in both analyzed subpopulations. These demographic analyses together with the observed unimodal pattern of mtDNA mismatch distributions (Figure 2) led us to infer presumably episodes of recent population expansions. The non-significant Harpending’s index (*r*) as well as the sum of squared deviations (SSD) between expected and observed distributions of pairwise differences would be supporting that assumption.

**Table 1:** Statistical parameters of Andalusian mtDNA control region sequences

	**Western Andalusians (Huelva)**	**Eastern Andalusians (Granada)**
N	158	121
K (%)	104 (65.82%)	95 (78.51%)
P	114	114
*H* (SD)	0.9856 (±0.0046)	0.9917 (±0.0035)
*D* (SD)	0.0113 (±0.0058)	0.0090 (±0.0047)
*M* (SD)	9.1776 (±4.2431)	7.3161 (±3.4479)
*SSD*	0.0017 (ns)	0.0013 (ns)
*r*	0.0028 (ns)	0.0033 (ns)
*D*	-1.71*	-2.11**
*F*_*s*_	-24.39***	-24.80***

K: number of different sequences and percentage of sample size in brackets; P: number of polymorphic sites; *H*: haplotype diversity and standard deviation; *D*: nucleotide diversity; *M*: mean number of pairwise differences; *SSD*: sum of squared deviations between the observed and expected mismatch distribution; *r*: Harpending’s raggedness index; *D* and *Fs* are, respectively, the Tajima's and Fu’s tests of selective neutrality.

ns: non-significant; *P < 0.05; **P <0.01; ***P < 0.001.

The mtDNA haplogroup composition of Andalusians (Table 2) revealed a typical west European example of Eurasian haplogroups (R0, HV, H, J, T, U w/o U6, K, N1, N2, and X) with proportions of 85.4% in the west (Huelva: n = 135 out of 158) and 96.7% in the east (Granada: n = 117 out of 121) of region. Even so, a significant genetic differentiation was observed, either by comparing control-region sequences (*F*_*ST*_ = 0.011; *P* = 0.000) or by considering the whole mtDNA haplogroup composition by adding coding-region information (*F*_*ST*_ = 0.025; *P* = 0.000). In support of this, the value χ^2^ = 41.54, df = 13, *P* = 0.000, was highly correspondent to the above mentioned *F*_*ST*_ values. The analysis of the corrected typified residuals revealed that the significant contributors (95% C.I.) to the genetic differentiation in the geographic distribution pattern for mtDNA diversity in the region were haplogroups K, H, U6, N1 and L.

**Table 2:** mtDNA lineages detected in contemporary Andalusian populations

**Haplogroup**	**Western Andalusians (Huelva)**		**Eastern Andalusians (Granada)**	
	**N**	**%**	**N**	**%**
***R0:***	***2***	***1.27***	***0***	***0.00***
R0a	2	1.27	0	0.00
***HV:***	***7***	***4.43***	***10***	***8.26***
HV0	5	3.16	4	3.31
HV0a	2	1.27	6	4.96
***H:***	***52***	***32.91***	***61***	***50.41***
H*	15	9.49	24	19.83
H1*	27	17.09	19	15.70
H1a	1	0.63	0	0.00
H1b1	0	0.00	1	0.83
H2a	1	0.63	1	0.83
H3	4	2.53	7	5.79
H5	2	1.27	8	6.61
H6	2	1.27	1	0.83
***J:***	***10***	***6.33***	***9***	***7.44***
J1*	8	5.06	7	5.79
J1b1	2	1.27	0	0.00
J2	0	0.00	2	1.65
***T:***	***10***	***6.33***	***11***	***9.09***
T*	0	0.00	3	2.48
T1	1	0.63	0	0.00
T2	9	5.70	8	6.61
***U(xU6):***	***25***	***15.82***	***15***	***12.40***
U2e	2	1.27	0	0.00
U3*	0	0.00	1	0.83
U3a	7	4.43	0	0.00
U4	3	1.90	0	0.00
U5*	0	0.00	1	0.83
U5a	2	1.27	2	1.65
U5b	11	6.96	10	8.26
U7	0	0.00	1	0.83
***K:***	***18***	***11.39***	***2***	***1.65***
K1	16	10.13	2	1.65
K2a	2	1.27	0	0.00
NR*:	***0***	***0.00***	***2***	***1.65***
NR*	0	0.00	2	1.65
***N1:***	***0***	***0.00***	***4***	***3.31***
N1b	0	0.00	2	1.65
I*	0	0.00	2	1.65
***N2:***	***6***	***3.80***	***2***	***1.65***
W*	6	3.80	2	1.65
***X:***	***5***	***3.16***	***1***	***0.83***
X2*	1	0.63	1	0.83
X2b	4	2.53	0	0.00
***U6:***	***14***	***8.86***	***2***	***1.65***
U6a	12	7.59	2	1.65
U6b	1	0.63	0	0.00
U6c	1	0.63	0	0.00
***M:***	***0***	***0.00***	***1***	***0.83***
M1	0	0.00	1	0.83
***L:***	***9***	***5.70***	***1***	***0.83***
L1b	4	2.53	0	0.00
L2a	2	1.27	0	0.00
L2b	3	1.90	0	0.00
L3	0	0.00	1	0.83
Total Eurasian lineages [R0, HV, H, J, T, U(xU6),K, NR*, N1, N2, X]	135	85.44	117	96.69
*Total North African lineages [U6, M1]*	14	8.86	3	2.48
*Total Sub-Saharan lineages [L1, L2, L3]*	9	5.70	1	0.83

Main haplogroups and frequencies are represented in bold italics.

### The influx of mtDNA lineages into Andalusians

#### ***Eurasian haplogroups***

Almost half of the western European matrilineal gene pool is composed of mtDNA lineages and sub-lineages that arise from the parental clade R0. A descendent lineage, haplogroup H, covers around 40-50% of the total mtDNA variation in Europe, with a decreasing cline towards the east and the south of the continent [15,26,27]. In the present study, Huelva Andalusians registered remarkably lower frequencies (32.9% of the total mtDNA variability) of H when compared with the proportion (50.4%) found in Granada (see Table 2). Haplogroup H shows higher values in northern Spain (~60%) [14,23].

Sub-haplogroups H1 and H3 register frequency peaks in the Iberian Peninsula, a scenario that has been traditionally interpreted in terms of mtDNA signals of a post-glacial major population expansion (~15 kya) from the Franco-Cantabrian refuge to northeast Europe [15,26]. These lineages are commonly found in western and eastern Andalusia (Huelva, H1: 17.7% and H3: 2.5% and Granada, H1: 16.5% and H3: 5.8%) as well as along the Mediterranean coast of Africa [15,28-30]. Moderate frequencies of H1 have been recorded in some Middle Eastern populations where H3 is almost absent [27].

Sub-clade H5 presents a rather high frequency (6.6%) in Granada sample when compared to their relatives from Huelva (1.3%) and other Iberian populations (2-4%, [19]). Among the whole mtDNA sequences of Granada Andalusians the proportion of H unclassified (i.e. H*, only polymorphism 7028 confirmed) is higher (~20%) than those found in other previously analyzed Iberian and North African samples [26,29,31,32]. It is worth noting that H* is the most commonly represented cluster within H in the Near East, a fact that could indicate that the paraphyletic group arose in this area and spread later over Europe [26].

The incidence of the sister clades J and T, both derived from R, were similar in the studied Andalusian samples although interesting diversification and differences in their distribution were observed when analyzing J and T phylogeny. Sub-haplogroup T2 comprises over 80% of the total T-lineages in western Europe [33], and it closely agree with that found in our study (in Huelva, 90.0% of T samples were T2, while we found 72.7% in Granada). Comparable scenarios have been observed in other neighboring coastal European Mediterranean populations (e.g. Southern Peninsular Italy, [34]) with the exception of Moroccan Berbers [31] and Egyptians [35] among which T1 sub-lineage is the most frequent.

mtDNA J sub-lineages J1b1a (np 242), J2a1a (nps 215, 16145, 16231) and J2b1a (np 16278) were detected among Andalusians. The two first ones (J1b1a, J2a1a) have been considered as signals of major expansions occurring from the Near East towards Europe in the Late Glacial period (~16-12 kya). The latter (i.e. J2b1a) is thought to be indeed an almost exclusive mtDNA marker of Europeans [33].

Our data revealed far higher frequencies of U/K clade in Huelva (36.1% of total U/K; 27.2% excluding U6) than in Granada (15.7% U/K; 14.1% excluding U6), contrasting also with the range of variation found in other previously studied Iberian samples (10-20%). Haplogroup U is a complex, ancient cluster that shows a heterogeneous distribution of its sub-clades across Eurasia [36]. In Andalusia region, U(×U6) yielded values ~14% with a differential presence of U2, U3, U4, U5 and U7 in the west and east of region. U5 is one of the most frequently U-lineages among modern European populations (7% on average, [37]). Accordingly, in Andalusia U5 (specifically U5b) covers most of variation of haplogroup U (7.0% out of 15.8% in Huelva and 8.3% out of 12.4% in Granada). Interestingly, U5b do not account for more than 10% in European Mediterranean populations and it is virtually absent in North Africa and Middle East [38].

Some members of the earliest branches of macrohaplogroup N (N1 and N2), and other phylogenetically related haplogroups such as X (X2) have been found in the whole studied Andalusian sample with frequencies <5%. Haplogroup N1 (N1b1), which is quite rare in Europeans but very frequent in Jews (~10% [39]), has also been detected in Granada Andalusians.

The incidence of haplogroup K in Huelva (11.4%) constitutes a distinguishing trait of its mtDNA gene pool, resembling the observed values (11-15%) found in Berbers from Tunisia and Egypt [31,40]. However, in other Iberian samples the frequencies of K (5-7%) agree with that found in most European populations [7,14,41]. The highest frequencies of Hg K are registered in Ashkenazi Jews (30% [39]). Even so, Ashkenazi Jews are rare and very recent in Spain.

#### ***African haplogroups***

Haplogroups U6 and M1 are considered female genetic markers of current northern African population, mainly along the Maghreb, and more eastern African areas [13,42-44]. In a European frame, patchy distributions of these haplogroups, together with L (mitochondrial lineage linked to sub-Saharan Africa) have been observed, although frequency peaks are reached in the Iberian Peninsula [6,10]. Western Andalusians (from Huelva, present study) register the highest frequencies (14.6%) of African lineages reported until now in the Iberian Peninsula and all over the European continent. The corresponding proportion in eastern Andalusians (Granada sample) was much lower (3.3%).

U6 appears in the Iberian Peninsula with frequencies between 0.5-5% [24,41]. In northwestern Africa, this lineage is relevant both in frequency and diversity (e.g. Moroccan Berbers, 11%, *H* = 1.000 ± 0.096 [31]; Tunisian Arabs, 8%, *H* = 0.833 ± 0.222 [45]). U6 sequences in Western Andalusians from Huelva are present over the Iberian range (~9%), being also are characterized by a high gene diversity value (*H* = 0.890 ± 0.060). Among the derived branches emerging from U6 clade, U6a is the most prevalent, widespread and diverse, reaching a frequency of 7.6% in Huelva province. Furthermore, the diversification of Hg U6 in western Andalusia (present study) is also extended to U6b and U6c lineages which display a more limited and fragmented phylogeography when compared to U6a [13,17]. Mitochondrial lineage M1 was only found in one individual from Granada sample (0.83%) mirroring the pattern detected in the Peninsula where this haplogroup is absent or recorded at levels lower than 1%.

Sub-Saharan L haplogroup is present in western Andalusia (Huelva: 5.7%, n = 9) through their derived clades L1b, L2a and L2b. Comparatively, only one individual L (L3h1b) was detected in the Granada sample. Lineage L2a is the most common and widespread mitochondrial haplogroup across the African continent [46] whereas L1b is concentrated in western and central Africa [47,48].

**Figure 3:**
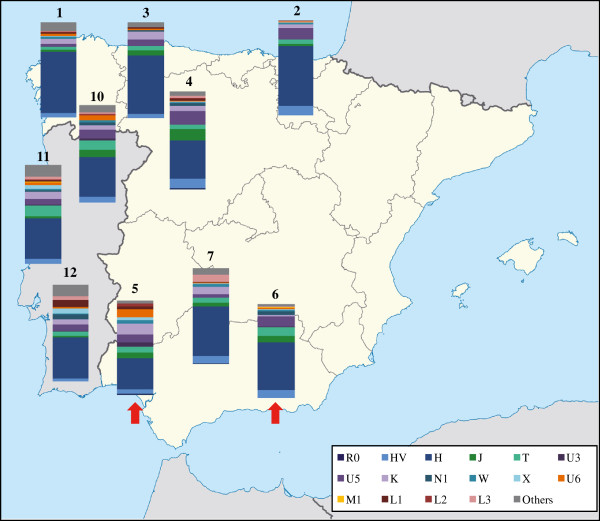
**mtDNA haplogroup profiles registered in some populations of the Iberian Peninsula.** The two Andalusian subpopulations studied here are marked with a red arrow. Codes are as in Additional file 3.

An updated graphical overview of the geographical variation of mtDNA profiles, found in earlier surveys of mainland Iberians, is given in Figure 3.

### Reconstructing Andalusian mtDNA sequence trees

Additional file 2 shows the gene tree built on the different mtDNA haplotypes observed among the analyzed Andalusian samples. As expected, a large number of haplotypes shapes the major cluster H (including H* and derived lineages) which presents a fair star-like phylogeny. Even though the rest of haplogroups did not show apparently a defined structure in the general phylogenetic network, the sequences belonging to L1b, L2b and U6a lineages are characterized by a high number of mutational steps from the rCRS (in the tree topology, the deepest and longest branches) with the exception of the branch length associated to L3h and U5b (see Additional file 1). These findings would be coherent with the early coalescence ages estimated for these haplogroups, and therefore related to its genetic history [42,46,49,50].

**Figure 4:**
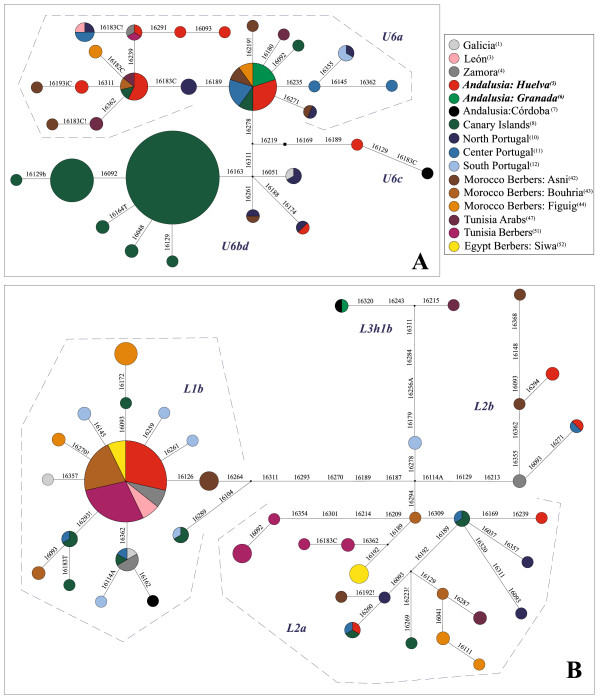
**Specific median-joining networks for lineages U6 (A) and L (B).** Control region sequences (HVS-I) showing relationships between Iberians and North African population have been used. Superscript numbers in legend refers to Additional file 3 population codes.

Depiction of control region haplotypes (HVS-I) of lineages U6 and L in a set of Iberian, Canarian and North African populations yielded different branch topologies. In the U6 network (Figure 4A) the sub-clade U6a (49.5%, 47 out of 95 mtDNA U6 samples) seems to be structured in two sub-clusters each showing a star-like phylogeny. TMRCA estimate for U6a (ρ = 1.28; σ = 0.63) disclosed an age of 21.29 ± 10.50 kya. The modal haplotype (on the right side), with 10 occurrences, is distinguished by transitions 16172-16219-16278 and comprises 5 mtDNA sequences from Andalusians (Huelva and Granada) together with Portuguese [41], Canarian [51] and Moroccan Berber samples [31]. The second large U6a node (16172-16183C-16189-16219-16278) has an outstanding contribution of Huelva Andalusians together with single samples from Tunisians Arabs, Moroccan Berbers and Canary Islanders. In a lesser extent, other derived rising haplotypes are common in two or more populations used in the analysis. Apart from U6a, U6 also comprises U6bd, being represented in the present network mainly by the autochthonous Canarian sub-lineage U6b1 (dark green color) [13] and the rare and less diversified sub-clade U6c, which is occupied by the punctual presence of single haplotypes from Huelva and Córdoba (the Caliphate capital town).

The haplogroup L network (in Figure 4B) also branches into different sub-clades, yet revealing a different structure than that obtained for U6. L1b shows a star-like phylogeny with the central node (the largest in the network) including 14 mtDNA sequences from 6 populations, being significantly represented Huelva Andalusians and two Berber samples (from Morocco and Tunisia). The age calculated for this lineage is 18.13 ± 5.49 kya (ρ = 1.09; σ = 0.33). Sub-clade L2a shows, by far, a dissimilar situation since it presents high doses of complexity and diversification. L2b is only represented by a central branch being occupied by Castilian and Berber related haplotypes and two tips belonging to two single individuals from Huelva and central Portugal. The rare L3h1b sub-lineage constitutes a peripheral branch within the network.

### Population genetic structure

We computed an AMOVA based on the entire population dataset reported in Additional file 3. Some results (not shown here) would indicate that mitochondrial DNA genetic differences among Iberian and the rest of geographic groups (marked by fixation indices *F*_*CT*_) were significant, except in the case of Iberia/Europe comparisons (0.08% of variance, *P* > 0.050). Interestingly, the extent of significance of *F*_*CT*_ between Andalusia and North Africa is less intense (*F*_*CT*_ = 0.029, *P* < 0.050) than when comparing the Iberian Peninsula as a whole to North Africa (*F*_*CT*_ = 0.041, *P* < 0.001).

**Figure 5:**
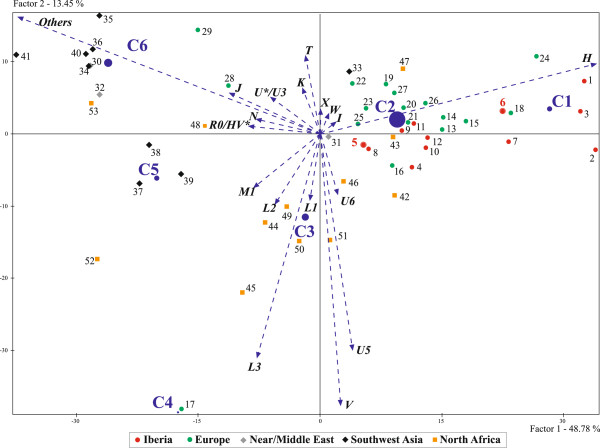
**Hierarchical Cluster Analysis (HCA) of 53 populations based on their mtDNA diversity.** The haplogroups used here are marked with arrows (vectors). Populations are indicated with numbers as in Additional file 3.

Figure 5 shows a Hierarchical Cluster Analysis (HCA) based on the same set of populations included in Additional file 3. Factors 1 and 2 account for 62.2% of the total variance, and, when considering the first four factors, an 81.1% of the variance is explained. The multivariate analysis provided six clusters with H, U5, V, L3 and “*Others*” lineages significantly defining the population topologies. When the inertia decomposition on the first two factors is computed, the quotient “Inertia interclusters/Inertia total” equals 0.843. This result points out that a high percentage of the data variation (84.3%) is explained by the six clusters.

Cluster 1 (C1) is strongly influenced by high frequencies of haplogroup H (cluster mean = 0.571; *P* = 0.000). Andalusian samples from Granada (#6) and Córdoba (#7) are within this cluster. It is worth noting that northern Spanish populations, such as Galicians (#1), Basques (#2) and Castilian-Leonese (#3) are positioned in the right extreme of the plot, due to the prominence of lineage H in these populations.

Cluster C2 is also significantly determined by haplogroup H (cluster mean = 0.386; *P* = 0.002) but in a lesser extent than C1. It groups a high number (n = 23) of populations: seven Iberians [including Huelva (#5) and 3 Portuguese samples (#10, #11, #12)], other nine from Mediterranean Europe and Middle East, and from continental Europe and North Africa. The genetic position of Huelva sample (#5) at the lower right quadrant of the bidimensional space, and its proximity to Canary Islands (#8) might be well interpreted by the relevant weight of haplogroup U6 in its mtDNA pool. Noteworthy, genetic affinity is found between Castilians from Zamora (north-central Peninsular Spain, #4) and northern Portuguese (one of their closest geographical neighbors, #10). This finding would be pointing out ancient admixtures before the emergence of the Portugal kingdom in the 12^th^ century.

C3 is significantly characterized by high frequencies of the typically African mtDNA clades: L3 (cluster mean = 0.172; *P* = 0.000), L2 (0.089; *P* = 0.000), L1 (0.051; *P* = 0.000), U6 (0.045; *P* = 0.002), and M (M1) (0.052; *P* = 0.013) and it coherently encompasses 7 populations from North Africa. Finally, cluster C5 groups southwestern Asia samples, determined by haplogroups R0, M, HV*, N and K, whereas C6 is mostly shaped by populations from the Middle East and other neighboring populations as Syria (#32), Iraq (#34), Yemen (#41) and Egypt (#53); the haplogroups included in the category “*Others*” (lineages not frequently found in western Europe) are defining this last cluster. We also run an additional HCA plot (not shown) excluding populations from clusters C4, C5 and C6. The population topologies keep the same pattern that the original HCA and no improvement of the visual interpretation of data was reached.

## Discussion

The mtDNA genetic pool of the Andalusians from Huelva and Granada has revealed a wide spectrum of haplogroups of different continental origins. The lower frequencies of Eurasian markers, together with the higher incidence and greater diversification of African maternal lineages among Huelva Andalusians –when compared to its relatives from Granada and other Iberian populations– constitute relevant findings previously unknown on the characteristics of mtDNA composition within Andalusia and the Iberian Peninsula, indicating indeed a female population substructure. Therefore, Andalusia must not be considered a single, unique population.

In the HCA of Figure 5 is observed that the two studied Andalusian populations belong to different clusters. Interestingly, Huelva is positioned in the central region of the bidimensional space, suggesting a varied mix implying populations from central, eastern Mediterranean and the Maghreb. Moreover, when analyzing the tree topology drawn from the HCA (data not shown), the first split separates the easternmost populations (Arabs, Egyptians, Caucasians and Iranians) from the rest of European and northwestern African populations. This split suggests that the extent of the admixture between those eastern populations out of their area was low, and consequently the East Mediterranean long coast between Egypt and Tunisia has been a rather impermeable barrier to mtDNA gene flow.

Migrations must be defined by their direction, being it determined by source and sink populations as well as by the time during which movement took place. The relationship between source and sink population sizes, and the distribution of their gene frequencies are important factors for the migration to leave a distinguishable genetic signal. The number of individuals shaping every migration must reach a moderate size, and although lineages may extend or disperse in more than one migration, the number of such movements must be limited, even in those territories in which migrations coming from so diverse origins have taken place, as is the case of the Iberian Peninsula. In the Maghreb, such number could be smaller than in Iberia, due to its geographical position. Furthermore, migrations among spatially distant regions must have occurred with minor intermediate steps to be genetically effective. The plausible timescale for those human movements should be posterior to those estimated for TMRCA of each lineage, taking into account lineage maturation time to be long enough to reach a sufficient frequency in the source population. Thus, a moderate number of emigrants might ensure a successful reproduction of such marker in the new settlement or host population. Regarding maternal lineages, those movements should imply women, which limit even more the final effective migration number.

Based on a genome-wide analysis, Botigué et al. [52] inferred elevated shared African ancestry in Iberia, which can be traced to populations in the North African Maghreb, hypothesizing that the higher diversity in southwestern Europe is a substantial contribution of recent migrations from North Africa. However, they also showed (Figures 1 and 2) a significant influence of Near East populations in the Iberian Peninsula. Our results, focused on mtDNA, reveal shared lineages among populations from Andalusia, North West Africa and other regions of the Mediterranean space.

The most adequate maternal lineages for studying migrations between the Iberian Peninsula and North Africa would be U6 and L. Nowadays, an interesting debate exists about the origins and spread of haplogroup U6. Some authors [42] argued that U6 was involved in an early dispersal (40–45 kya) from southwestern Asia to North Africa. Recently, other researchers [44] have proposed that some of the topologically earliest sub-clades of U6 may have been linked with the Iberomaurusian culture (20–9 kya), covering mainly Northwestern Africa.

**Figure 6:**
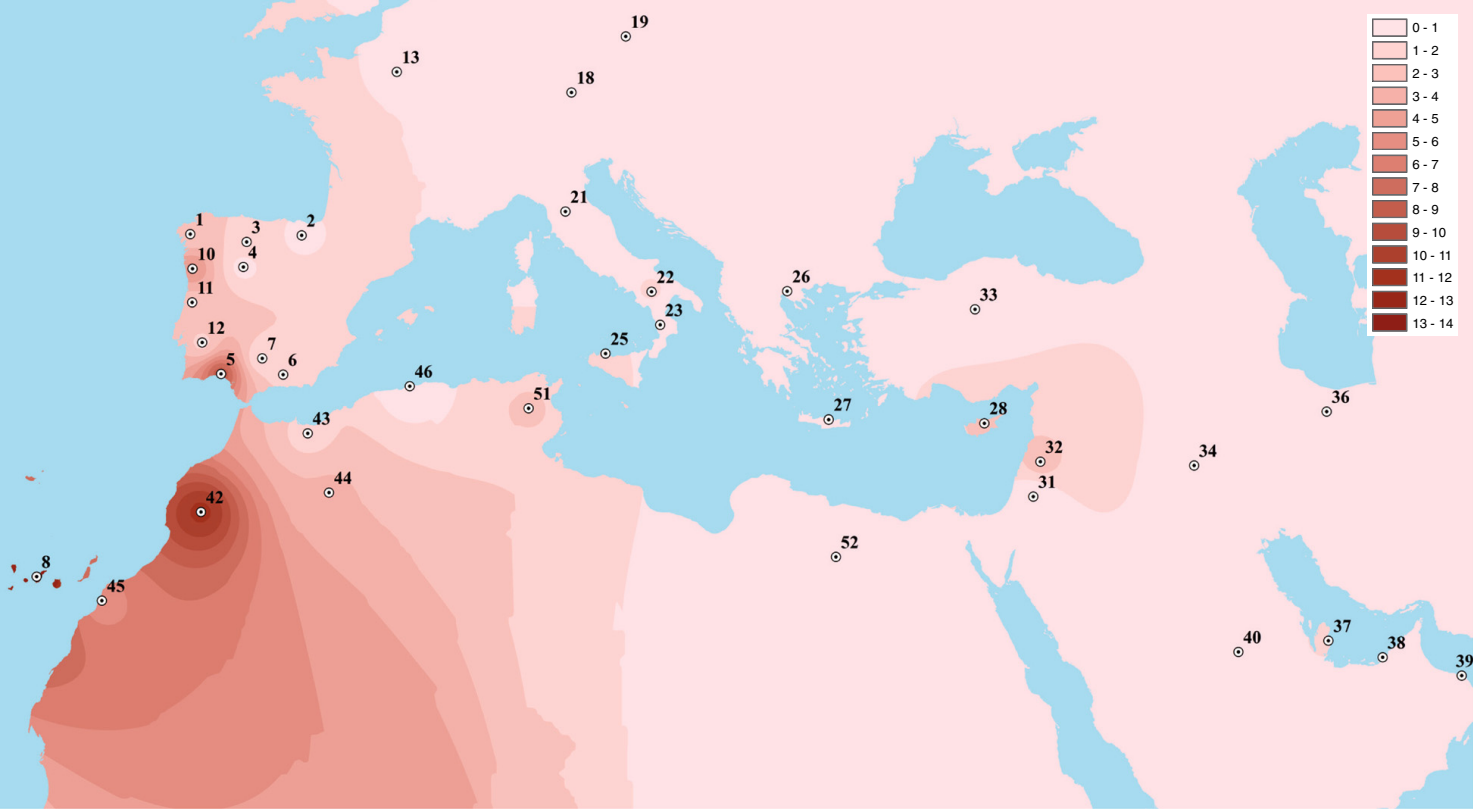
**Interpolation frequency (%) map of lineage U6 across Mediterranean basin.** See codes and references in Additional file 3.

Figure 6 displays a surface map with the geographic variations of U6 haplogroup around the Mediterranean. Whilst this kind of analysis only permits to broadly visualize the phylogeography of U6 (and not migration times) it specially highlights how the Atlantic façade of Morocco –including the neighboring Canary Islands– concentrates high frequencies of this lineage, and how its spatial pattern fairly exhibits a rather soft decreasing geographical continuity from Morocco in the north direction, reaching the whole western belt of Iberian Peninsula with the most perceptible frequencies in the territory of Huelva (present study).

Median-joining trees shed some light over the relationship between shared lineages (haplotypes) as well as their shape and position. In the U6 lineage network (see Figure 4A) the abundant Canary samples –except for two of them– belong to haplogroup U6b1. The high frequency of such archipelago-specific lineage would not have influenced the migrations occurred between Europe and Africa, due to its small population size, the late colonization time (2.5 kya) of the Canary Islands, and the relatively late evolutionary age (~13 kya) of U6b [44]. U6c, with an estimated coalescence age of 11 kya [44,50] comprises only two Andalusian individuals, with no trace of African influence. Most of the non-Canary individuals belong to U6a, which shows two major nodes, both connected by two mutations, where Iberian lineages (n = 11) predominate over the North African ones. U6a tip branches show indeed no derivations through North African lineages.

Muslim expansion has been widely cited as the main cause for gene flow between the Iberian Peninsula and North Africa, yet it might be only one of several interactions due to multiple coast-to-coast human movements. The bigger population size on the Iberian side would have presumably left a more noticeable genetic signature over North African population than the inverse (reciprocal) movements. The population size in the south of the Iberian Peninsula –information based on archaeological sites – was already relevant since Paleolithic onwards (see Figure 5 in [12]) and although no data on North African population size is provided it might have been presumably smaller using similar methodology. Considering these demographic findings along with the network structure and the abundance of U6a in southwestern Iberia, some sub-clades of this lineage could have their origin in the Iberian Peninsula from where it there would have expanded towards North Africa. The presence of U6 in Near East, with lower frequencies than in Africa, and also in Ethiopia would reflect some contacts across the sea in the past. The hypothesis of a terrestrial way along the coastal North African fringe seems less likely, provided the large distance and the absence of settlement areas supplied with enough food resources between Nile estuary and Tripoli region. These considerations indicate that repeated contacts between populations in these both opposite geographical locations in the past seem unlikely.

Some researchers (e.g. [53,54], among others) have proposed a sea route as the most probable way for Neolithic entrance in Iberia. This process was phased, using sea navigation and boats big enough to transport men, women, and the ‘*Neolithic package’* in a movement probably originated in the Gulf of Genoa. Similar methods may have been used between Andalusia and Morocco. Altogether, these events suggest that the interactions between Moroccan and Andalusian populations have been old, continuous, in both ways and with different origins.

Thereafter, during the colonial expansion on early 1^st^ millennium BC, there were some maritime contacts between eastern and western Mediterranean. These contacts continued during Carthaginian hegemony and the long-lasting Roman Empire rule. As a result of Diocletian’s administrative reorganization (late 3^rd^ century AD), northern Moroccan province *Mauretania Tingitania* was grouped together with peninsular provinces to form the *Diocesis Hispaniarum* from which Tangier region (Morocco) and Andalusia were the first territories to be Christianized at the beginning of the 4^th^ century. Therefore, it seems unlikely that U6a lineages located in the network core were the result of the posterior Islam expansion in Iberia, despite the multiple invasions occurred from the Maghreb to the Peninsula during this period. Interestingly, other new genetic data would support that hypothesis. The outstanding presence of European specific haplogroups in the maternal gene pool of contemporary northwestern African human populations may account for the occurrence of migrations from Europe, being the Iberian Peninsula an important source of that gene flow [28,30,32]. Thus, the Maghreb might have experienced genetic maternal flow from the European continent since ancient times across the sea.

In the sub-Saharan L macrohaplogroup network (Figure 4B), lineage L1b is characterized by the relevant case number contained in the core. According to [50], its estimated coalescence time is 9.7 kya, so its expansion out of Africa should have taken place during or after Neolithic age. The L1b star-like shape indicates a population expansion, and those migrations which contributed to shape it were not so recent, thus Muslim expansion or more recent migrations accounting for this would not be the main causal reason. Furthermore, the L1b central core is curiously composed by Berbers from a wide geographical area -ranging from Egypt to Morocco- and Spaniards from western provinces, from Huelva (in the southern corner) to León (in the northwest). Both Spanish territories were connected by the Roman road named *Via de la Plata* (“silver way”) which acquired a remarkable military and trade importance not only during Roman Empire, yet before and after it, since it connected important cities and mining deposits. This road might have permitted population movements from the south- and north-western Spain during long periods. These maternal lineages shared among western Spanish populations may correspond to women supporting military contingents or trading. Since Portuguese populations are absent from the L1b core, and all the Portuguese sequences belonging to this lineage are derived, Spanish and Portuguese genetic histories may have been rather different for this mitochondrial sub-haplogroup.

Lineage L2a, the second most represented in the network (coalescence age ~48 kya, [50]) shows no star-like shape. This haplogroup is composed by a Berber majority and some Iberians, and only few haplotypes are shared. In Iberia, these lineages may be associated to Islamic expansion, which penetrated up to North Portugal, rendering its relationship with recent slave trade unlikely. However, its relationship with slavery during Roman Empire or Islamic rule cannot be ruled out.

Some authors [6] pointed at the Atlantic slave trade (15^th^-19^th^ centuries) as the source of L lineages in Portugal, the main destination of black slaves in Europe. In this way, some authors have discarded a likewise input move for U6 and L haplogroups in the Peninsula, arguing that both mtDNA clusters show different geographic distributions and frequencies in autochthonous populations from Portugal and Spain. Some sub-Saharan genes coming from Ibero-America would have been introduced in Iberia as well. The presence and the relatively high frequencies of the African immunoglobulin allotype GM*1,17 23′ 5* in western Spanish populations has been partially interpreted in the same frame [4,55].

Migrations between Maghreb and Iberian Peninsula had to take place across the sea since Paleolithic and the transport of women implied the use of bigger watercrafts than those crewed only by men. Sea navigation might have been commonly practiced as a fishing technique, without losing sight of the land for safety. The shortest distance between Europe and Africa is located at the Gibraltar Strait, and such distance markedly increases as one move towards east, reaching an approximately steady separation of around 150 km. Winds and ocean currents are very variable and dangerous around the Strait, and an alternative though not excluding calmer route might have been those that connects Cape Three Forks (Morocco) with the Andalusian coast, between Malaga and Almeria, in which the small Alboran Island (approximately 36°N, 3°W) is halfway, and could be used as a stopover. Within this *Alboran route*, currents and winds go from Europe to Africa most of the year, and the high Rif and Sierra Nevada Mountains permit a permanent sight of land during the journey. So, it seems likely that the island was known among Paleolithic hunter-gatherers, due to its richness in sea mammals, big fishes and an extensive intertidal zone, probably used by humans, for its rich fauna food [56]. Furthermore, archaeological remains found in eastern Morocco (e.g. *the Taforalt harpoon*, [57]) or evidences of big-game fishing (in Nerja cave, Malaga) during the Upper Magdalenian (~12-10 kya) suggest the existence of contacts between the north and south of western Mediterranean shores.

## Conclusions

The obtained results underline the necessity of further research on genetic relationships in both sides of the western Mediterranean, using carefully collected samples from autochthonous individuals. Such studies should include different markers analyzed in a high genetic resolution, since migrations have been numerous in the region through a long period of the human history, presumably starting in Paleolithic times. Many studies have focused on recent North African gene flow towards Iberia, yet scientific attention should be now directed to thoroughly study the introduction of European genes in northwest Africa across the sea, in order to determine its magnitude, timescale and methods, and to compare them to those terrestrial movements from eastern Africa and southwestern Asia. The African origin of modern *Homo sapiens* does not imply that peopling of the northwestern side of that continent took place from the inside exclusively.

## Methods

### Populations and samples

We have analyzed 279 mtDNA sequences from healthy, unrelated individuals of both sexes with geographic origins in Huelva (n = 158) and Granada provinces (n = 121). Further details on geography, history and demography of the studied populations can be found in [4,9].

For sampling processes, which must be considered as a critical activity in this kind of works, demographic stability and historical criteria were taken into account in the selection of the localities; touristic coastal areas, as well as the capital cities of the provinces were discarded. Each participant was informed about the aims of this research and full informed consent was obtained from all donors. Maternal ancestry of donors was recorded for a minimum of three generations born in the same province. The analyzed subjects were selected from a bigger sample collected during 2004–2008 by members of this research team, helped by local health workers. Andalusian DNA stock samples are available in our Laboratory of Molecular Anthropology (Department of Zoology and Physical Anthropology, Complutense University of Madrid). The present research project and protocols have been officially approved by the Bioethics Committee of the Complutense University of Madrid.

### DNA extraction

Genomic DNA was isolated from blood samples (≈5-7 ml) by means of a standard proteinase-K digestion followed by phenol-chloroform extraction and ethanol precipitation.

### mtDNA molecular analysis

For each sample, mtDNA hypervariable region I (HVS-I) and part of HVS-II (≈820 bp) were amplified using primer pairs F15973 and R296 (for more details, see [31]). Sequence reactions were carried out using the BigDye Terminator v3.1 Cycle Sequencing Kit (Applied Biosystems) and run in an automatic Sequencer ABI PRISM 3730 (Genomics Unit, Complutense University of Madrid). Both DNA strands were sequenced using primers F15973 and R296, and those samples harboring a homopolymeric cytosine stretch between nps 16148–16193 were sequenced twice in each sense in order to get the consensus sequence. In addition to the control region sequencing, 47 coding region informative SNPs were surveyed for haplogroup assignment either by sequencing or by PCR-RFLPs (see Additional file 1).

There were two individuals from Granada that could not be classified in any concrete lineage, neither by control sequences nor by RFLPs. Therefore, they were categorized as NR* since we could only confirm the polymorphism 10873.

### Statistical and phylogenetic analyses

mtDNA control region sequences obtained were aligned by using BioEdit v.7 software [58] and only confirmed deviations from the revised Cambridge reference sequence (rCRS, [59]) were considered. Haplogroup assignment (using control region haplotype and coding region SNPs) was based on the updated phylogenetic data available in Phylotree website (mtDNA tree Build 13, [60]).

Within-population genetic diversity parameters of the studied populations were calculated. To check deviations from selective neutrality, and thus to assess evidence for presumable population expansion, we employed the Tajima’s (*D*) [61] and Fu (*F*_*S*_) [62] statistics. Normal distribution of pairwise differences was evaluated by the use of Harpending’s raggedness index (*r*) [63] and by the sum of squared deviations (SSD) between the observed mismatch distribution and the expected one under demographic expansion [64]. Molecular pairwise *F*_*ST*_ indexes between the two Andalusian populations were also calculated (using sequence information, as well as mtDNA haplogroup composition), and their significance was evaluated with a nonparametric permutation test. Previous tests were performed using Arlequin 3.5 software [65]. In addition, a χ^2^ test was performed and the corrected typified residuals were analyzed to assess differences in haplogroup profiles (IBM SPSS Statistics 19).

To construct maternal genealogies, a median-joining network [66] was inferred from the haplotypes found in Huelva and Granada population samples by using the program Network 4.5 (http://www.fluxus-engineering.com). The most parsimonious tree was constructed by hand, and confirmed by the Network MP-calculation option. We followed the weighting scheme proposed by [67] for all nucleotide positions used in the present analysis (nps 16023–264). Following the same methodology, specific networks for African mtDNA lineages U6 and L were constructed, using a set of Iberian (including Canary Islands) and North African populations and based on control region (HVS-I) haplotypes. We calculated values of internal U6 gene diversity using population sequence information from literature [31,45]. Moreover, we estimated TMRCA ages for some selected African sub-clades, using ρ statistic values converted into years by using the corrected mutation rate for HVS-I proposed by Soares et al. [50].

Likewise, dataset of 53 selected populations drawn from the literature was used for performing an Analysis of Molecular Variance (AMOVA) [68]. This test was based on haplogroup frequencies and calculated by means of Arlequin 3.5 [65]. On the same population database (see Additional file 3), a Hierarchical Cluster Analysis (HCA) –based on mtDNA haplogroup frequencies– was used for evaluating population relationships by the use of SPAD software (*Système Portable pour l’Analyse des Données*, [69]). This multivariate analysis is often employed for constructing genetic maps because of its high level of statistical resolution. The clusters established in a hierarchical and progressive way depend on the distance among populations. In order to evaluate distance between clusters by minimizing the squared sum within each group, the Ward’s linkage algorithm is used. The HCA is performed on the basis of Euclidean distances.

Finally, the geographical distribution of lineage U6 in the Mediterranean space was represented by a surface interpolation map built with ArcGIS 10.1 (*Spatial Analyst Extension*). This contour map was constructed using *Inverse Distance Weighted* method (IDW), which assumes that the influence of the variable being mapped (haplogroup U6 frequency) decreases with distance from the sampled locations. We used a power of two for the analysis.

### Accession numbers

Sequences analyzed in the present study have been submitted to GenBank under accession numbers KJ169731 - KJ170009.

## Author’s contributions

RC designed the sampling process together with JNR and collected the blood samples in the Andalusia region. CLH and GR carried out molecular analysis to genotype Andalusian samples. CLH, PC and GR worked together in the genetic and statistical data analysis. RC and CLH took the responsibility of writing the paper. JMD and AN did interesting suggestions while preparing the manuscript and helped in the coordination of the study. All authors contributing to this study read and approved the final manuscript.

## Supplementary Material

Additional file 1: Table S1 Mitochondrial molecular characterization of the 279 analyzed Andalusian samples. Transversions are indicated with the explicit base change. Insertions are marked with “i”, deletions with “d” and heteroplasmy with “Y” (C/T). Coding region polymorphisms are reported in the hierarchical order as tested. GenBank accession numbers for control region sequences are included. Click here for file

Additional file 3: Table S2 A set of selected human populations used for mtDNA comparative analyses. The geographic regions established are *IB*: Iberia, *EUR*: Europe, *NME*: Near/Middle East, *SWA*: Southwest Asia, *NAF*: North Africa. Click here for file

Additional file 2: Figure S1 Median-joining network showing the phylogenetic relationship of the 197 Andalusian mtDNA haplotypes. Haplogroups are shown in blue. The mutated positions reported in the branches refer to the reference sequence rCRS (Andrews et al. 1999). All polymorphisms are control region mutations, except those in red (coding region haplogroup diagnostic positions). Transversions are specified with the base change after the mutation. Suffix “i” indicates an insertion, “d” a deletion and “Y” heteroplasmy (C/T). Retromutations are indicated with an exclamation mark. Size of circles is proportional to the haplotype frequency. Huelva and Granada samples are marked in red and green, respectively. Click here for file
